# Effect of sitagliptin on blood glucose control in patients with type 2 diabetes mellitus who are treatment naive or poorly responsive to existing antidiabetic drugs: the JAMP study

**DOI:** 10.1186/s12902-016-0149-z

**Published:** 2016-12-01

**Authors:** Hiroshi Sakura, Naotake Hashimoto, Kazuo Sasamoto, Hiroshi Ohashi, Sumiko Hasumi, Noriko Ujihara, Tadasu Kasahara, Osamu Tomonaga, Hideo Nunome, Masashi Honda, Yasuhiko Iwamoto, for the JAMP Study Investigators, Akiko Sato, Akinori Yamashita, Akira Miyashita, Asako Kokubo, Atsuro Tsuchiya, Dai Hirohara, Daiji Kogure, Daijo Kasahara, Hideki Tanaka, Hideki Tanaka, Hideo Tezuka, Hiroyuki Kuroki, Jun Ogino, Kanu Kin, Kanu Kin, Kazuko Muto, Kazuo Suzuki, Keiko Iseki, Keita Watanabe, Kenshi Higami, Kenzo Matsumura, Kiyotaka Nakajima, Koki Shin, Kuniya Koizumi, Maki Saneshige, Makio Sekine, Makoto Yaida, Mari Kiuchi, Mari Mugishima, Mari Osawa, Masae Banno, Masahiro Yamamoto, Masatake Hiratsuka, Masumi Hosoya, Michika Atsuta, Mitsutoshi Kato, Miwa Morita, Munehiro Miyamae, Mutsumi Iijima, Naomi Okuyama, Nobuo Hisano, Norihiro Tsuchiya, Rie Wada, Rie Wada, Ryuji Momose, Sachiko Otake, Satoko Maruyama, Satoru Takada, Shigeki Dan, Shigeki Nishizawa, Shigeo Yamashita, Shingo Saneshige, Shinichi Teno, Shinji Tsuruta, Shinobu Kumakura, Sumiko Kijima, Takashi Kondo, Takeo Onishi, Taku Kudo, Tatsushi Sugiura, Toshihiko Ishiguro, Yasue Suzuki, Yasuhiro Tomita, Yasuko Takano, Yoshihisa Akimoto, Yoshiko Odanaka, Yoshimasa Tasaka, Yoshitaka Aiso, Yukiko Inoue, Yukinobu Kobayashi

**Affiliations:** 1 Department of Medicine, Medical Center East, Tokyo Women’s Medical University School of Medicine , 2-1-10 Nishiogu, Arakawa-ku, Tokyo 116-8567 Japan; 2 Department of Diabetes, Endocrine and Metabolic Diseases, Tokyo Women’s Medical University Yachiyo Medical Center, 477-96, Owada-shinden, Yachiyo-shi, Chiba 276-8524 Japan; 3 Internal Medicine, Suzuki Clinic, 2-10-14, Koyasu-machi, Hachioji-shi, Tokyo 192-0904 Japan; 4 Internal Medicine, Oyama East Clinic, 1-32-1, Ekihigashi-dori, Oyama-shi, Tochigi 323-0022 Japan; 5 Internal Medicine, Nishiyamado-Keiwa Hospital, 3247-1, Kounosu, Naka-shi, Ibaraki 311-0133 Japan; 6 Department of Medicine, Diabetes Center, Institute of Geriatrics, Tokyo Women’s Medical University, Shibuya Cross Tower 22F, 2-15-1, Shibuya, Shibuya-ku, Tokyo 150-0002 Japan; 7 Josai Hospital, 2-42-11, Kamiogi, Suginami-ku, Tokyo 167-0043 Japan; 8 Diabetes and Lifestyle Center, Tomonaga Clinic, Shinyon curumu Building 9F, 4-2-23, Shinjuku, Shinjuku-ku, Tokyo 160-0022 Japan; 9 Diabetes Center, Edogawa Hospital, Medical Plaza Shinozaki, SK Building, 7-15-12, Shinozaki-machi, Edogawa-ku, Tokyo 133-0057 Japan; 10 Nishikawa Clinic, 2-16-3, Towa, Adachi-ku, Tokyo 120-0003 Japan; 11 The Institute for Adult Diseases, Asahi Life Foundation, Asahiseimeisunaga building, 2-2-6, Nihonbashi Bakuro-cho, Chuo-ku, Tokyo 103-0002 Japan

**Keywords:** Sitagliptin, Diabetes mellitus, DPP-4 inhibitor, HbA1c, Glimepiride

## Abstract

**Background:**

To investigate the ameliorating effect of sitagliptin, a dipeptidyl peptidase-4 inhibitor, on blood glucose control in patients with type 2 diabetes mellitus who were previously untreated with or who have a poor responsive to existing antidiabetic drugs.

**Methods:**

Sitagliptin (50 mg/day) was added on to the pre-existing therapy for type 2 diabetes and changes in the glycated hemoglobin (HbA1c) level after 3 months of treatment were compared with the baseline and performed exploratory analysis.

**Results:**

HbA1c levels were significantly decreased after 1 month of treatment compared to baseline, with a mean change in HbA1c level from baseline of −0.73% (range, −0.80 to −0.67) in the entire study population at 3 months. Patients who received a medium dose of glimepiride showed the least improvement in HbA1c levels. The percentage of patients who achieved an HbA1c level of <7.0% significantly increased after 1 month of treatment, reaching 53.1% at 3 months. The percentage of patients who achieved a fasting blood glucose level of <130 mg/dL significantly increased after 1 month of treatment, reaching 50.9% at 3 months.

**Conclusions:**

Sitagliptin improved the HbA1c level and rate of achieving the target control levels in patients with type 2 diabetes mellitus who were previously untreated with, or poorly responsive to, existing antidiabetic drugs. Thus, sitagliptin is expected to be useful in this patient group. However, the additional administration of sitagliptin in patients treated with medium-dose glimepiride only slightly improved blood glucose control when corrected for baseline HbA1c level.

**Electronic supplementary material:**

The online version of this article (doi:10.1186/s12902-016-0149-z) contains supplementary material, which is available to authorized users.

## Background

The 2012 National Health and Nutrition Survey conducted by the Ministry of Health, Labour and Welfare of Japan (MHLW) estimated that approximately 20.5 million people were strongly suspected of having diabetes mellitus; these patients are considered the at-risk group of individuals in whom diabetes mellitus cannot be ruled out [1]. An increasing amount of patients strongly suspected of having diabetes mellitus are currently being treated with existing antidiabetic drugs (men, 65.9% and women, 64.3%) [1]. However, existing antidiabetic drugs have various drawbacks, including insufficient efficacy, short-lasting effect [2], body weight increase and inconvenient administration. Thus, new drugs that have a different mechanism of action and that show improved efficacy, safety and tolerability are required.

Dipeptidyl peptidase-4 (DPP-4) inhibitors exhibit antidiabetic effects by stimulating insulin secretion through highly selective inhibition of DPP-4, an enzyme that inactivates incretins such as glucagon-like peptide 1 and gastric inhibitory polypeptide via a mechanism different from that of conventional hypoglycemic drugs. Many reports have demonstrated the superior efficacy and safety of DPP-4 inhibitors [3–5], among which sitagliptin was the first to gain approval in Japan in 2009. Many reports of sitagliptin had published, but we have examined the additional effect of sitagliptin in 7 pre-existing therapy groups. Treatment of these patients often includes other antidiabetic medications because they often have diverse complications. The objective of this study is to investigate the ameliorating effect of sitagliptin on blood glucose control in patients with type 2 diabetes mellitus, who were previously untreated with, or who had a poor responsive to, existing antidiabetic drugs.

## Methods

### Study design

This open-label, central registration, multi-center, prospective observational study was conducted at the Tokyo Women’s Medical University Hospital and 69 collaborating institutions in Japan. Patients were enrolled from 1 January 2011 to 30 June 2013, and followed up until 30 June 2014. This study was conducted with the approval of the ethic committee of the Tokyo Women’s Medical University (UMIN000019154).

### Study subjects

The study subjects were male or female, 20 years of age or older, and outpatients with type 2 diabetes mellitus and inadequately controlled blood glucose levels (a glycated hemoglobin (HbA1c) level of ≥6.9% (52 mmol/mol) or a fasting blood glucose level of ≥ 130 mg/dL [6] during the observation period) after at least 1 month of receiving diet/exercise therapy or/and oral antidiabetic drug therapy.

The participants were treated with diet and exercise therapy, low-dose glimepiride (0.5–1 mg), medium-dose glimepiride (1.5–2 mg), biguanides, thiazolidines, α-glucosidase inhibitors or two or more of these drugs in combination during the observation period. And patients treated with more than 2 mg of glimepiride or other SUs were excluded from the study.

The all patients provided written informed consent before participation.

Patients who met any of the following criteria were excluded from the study: (i) history of severe ketosis, diabetic coma or pre-coma within the past 6 months; (ii) severe infection before or after surgical treatment, or serious external injury; (iii) pregnancy, possible pregnancy or lactation; (iv) moderate renal impairment (serum creatinine level ≥1.5 mg/dL in men and ≥1.3 mg/dL in women); (v) patients on insulin therapy; (vi) patients on treatment with rapid-acting insulin secretagogues; (vii) history of allergy to the ingredients of the study drug; and (viii) a medical reason that makes the patient unsuitable for participation in the study as judged by the investigator.

Treatment with (v) and/or (vi) were not covered by national health insurance at the time of setting the study protocol.

### Treatments

**Fig. 1:**
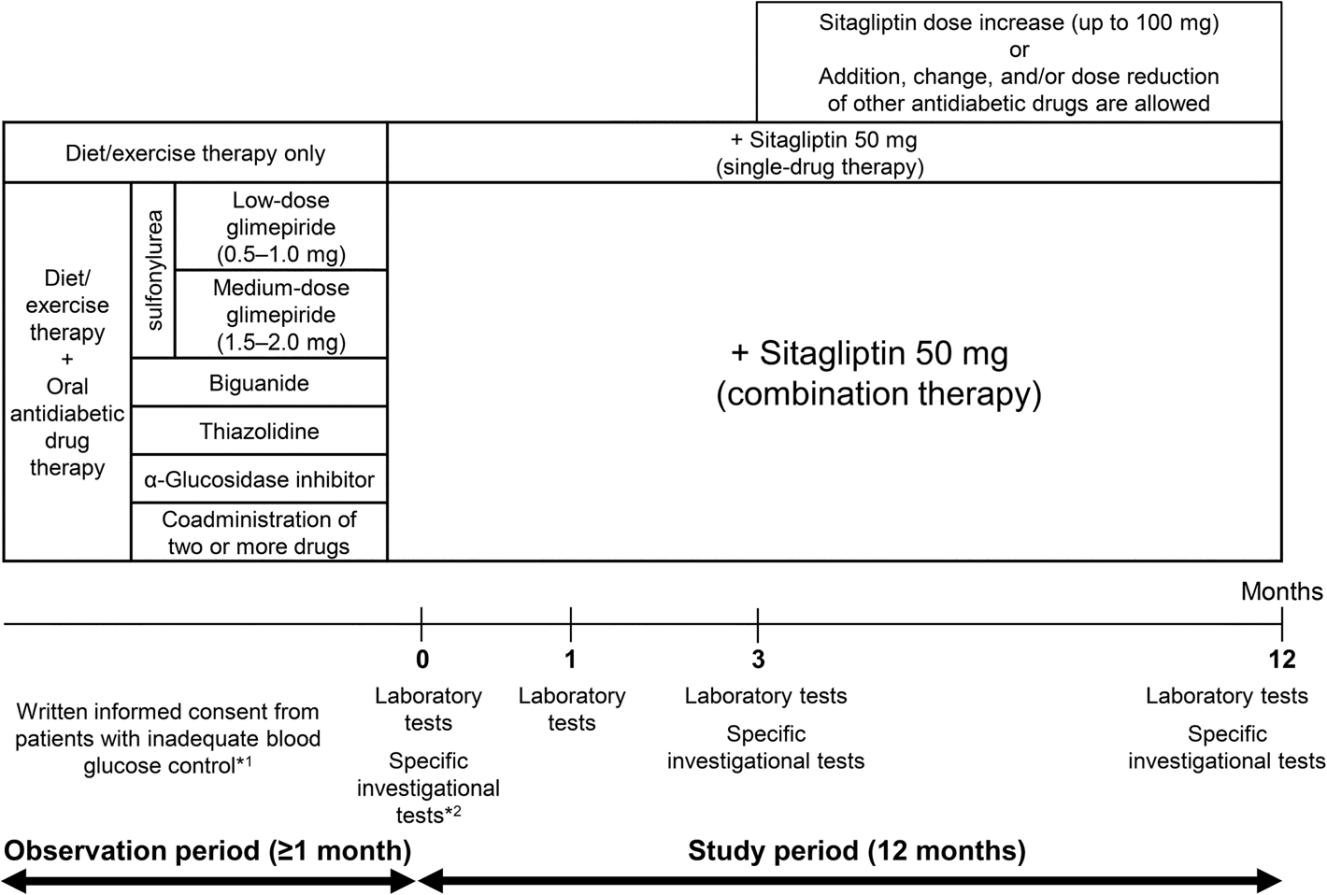
Study design. *^1^ Criteria for inadequate blood glucose control: HbA1c level ≥6.9% (52 mmol/mol) or fasting blood glucose level ≥130 mg/dL. *^2^ Specific investigational tests (optional): GA, 1.5AG, C-peptide, proinsulin-to-insulin ratio

The pre-existing therapy for type 2 diabetes were not changed during the observation period and entire study period (for the first 3 months after add-on sitagliptin). Thereafter, sitagliptin (50 mg) was administered once daily as a first-line treatment (single-drug therapy) or as an additional treatment (combination therapy; Fig. 1). During the 3-month period from the initiation of sitagliptin treatment (baseline), administration of sitagliptin was continued without the addition of any other drugs or dose increases. At 3 months, the sitagliptin dose was increased from 50 to 100 mg/day and other antidiabetic drugs were added, changed or discontinued at the investigator’s discretion. No restrictions were imposed on the use of drugs for treating concurrent diseases, but dose changes or the addition of new drugs was avoided whenever possible during the study period.

### Evaluation

All end points were defined in the study protocol prior to implementation of the study. The primary end point was the change in HbA1c level at 3 months from baseline. The secondary end points were as follows: the percentage of patients who achieved a normal HbA1c level (<7.0%, 53 mmol/mol [6]) and the percentage of patients who achieved a normal fasting blood glucose level (<130 mg/dL) at 1, 3 and 12 months after the start of treatment; changes in HbA1c from baseline at 1 and 12 months after starting treatment; and fasting blood glucose level, homeostatic model of assessment-β index and blood lipid levels (low-density lipoprotein cholesterol [LDL-C], triglycerides [TG], and high-density lipoprotein cholesterol [HDL-C]) at 3 and 12 months after starting treatment.

In addition, the HbA1c level at 3 months of treatment and the change in HbA1c level from baseline were calculated for each patient group classified according to the concomitant drug used. The approximate linear correlation between the baseline HbA1c level and the change in HbA1c level after 3 months of treatment was examined statistically using an exploratory approach. Multiple linear regression analysis was performed using the deviation from the correlation line as the dependent variable. Safety assessments included the incidence of adverse drug reactions and hypoglycemia during the study period.

At the start of the study in 2011, HbA1c values were expressed in The Japan Diabetes Society levels, the standard system in Japan, but were changed to National Glycohemoglobin Standardization Program (NGSP) system values at the end of the study in accordance with the “Report of the Committee on the Classification and Diagnostic Criteria of Diabetes Mellitus (Revision for International Harmonization),” issued by The Japan Diabetes Society [7]. Pursuant to the above change, the lower limit of inadequate blood glucose control was also changed. Therefore, patients with an HbA1c level of ≥6.9% (52 mmol/mol) were enrolled at the start of the study, but at the data analysis stage, the percentage of patients who achieved the target HbA1c level of ≥7.0% (53 mmol/mol) or <7.0% (53 mmol/mol) was calculated.

### Statistical analysis

Data analysis was performed using IBM SPSS Statistics 22.0 for Windows (IBM Japan, Ltd.). Observed values were analyzed using a paired *t* test, while a between-group comparison of changes was performed using analysis of variance. The chi-square test was used to analyze the percentage of patients who achieved the HbA1c target level, and correlation was evaluated using Pearson’s test. The factors that affect the fasting blood glucose-lowering effect were evaluated using simple and multiple regression analyses, with the significance level set at 5% (two-sided). Demographic characteristics are presented as the mean ± standard deviation (SD), and the observed values are presented as the mean ± standard error (SE). Changes are presented as the mean (95% confidence interval [CI]).

## Results

**Fig. 2:**
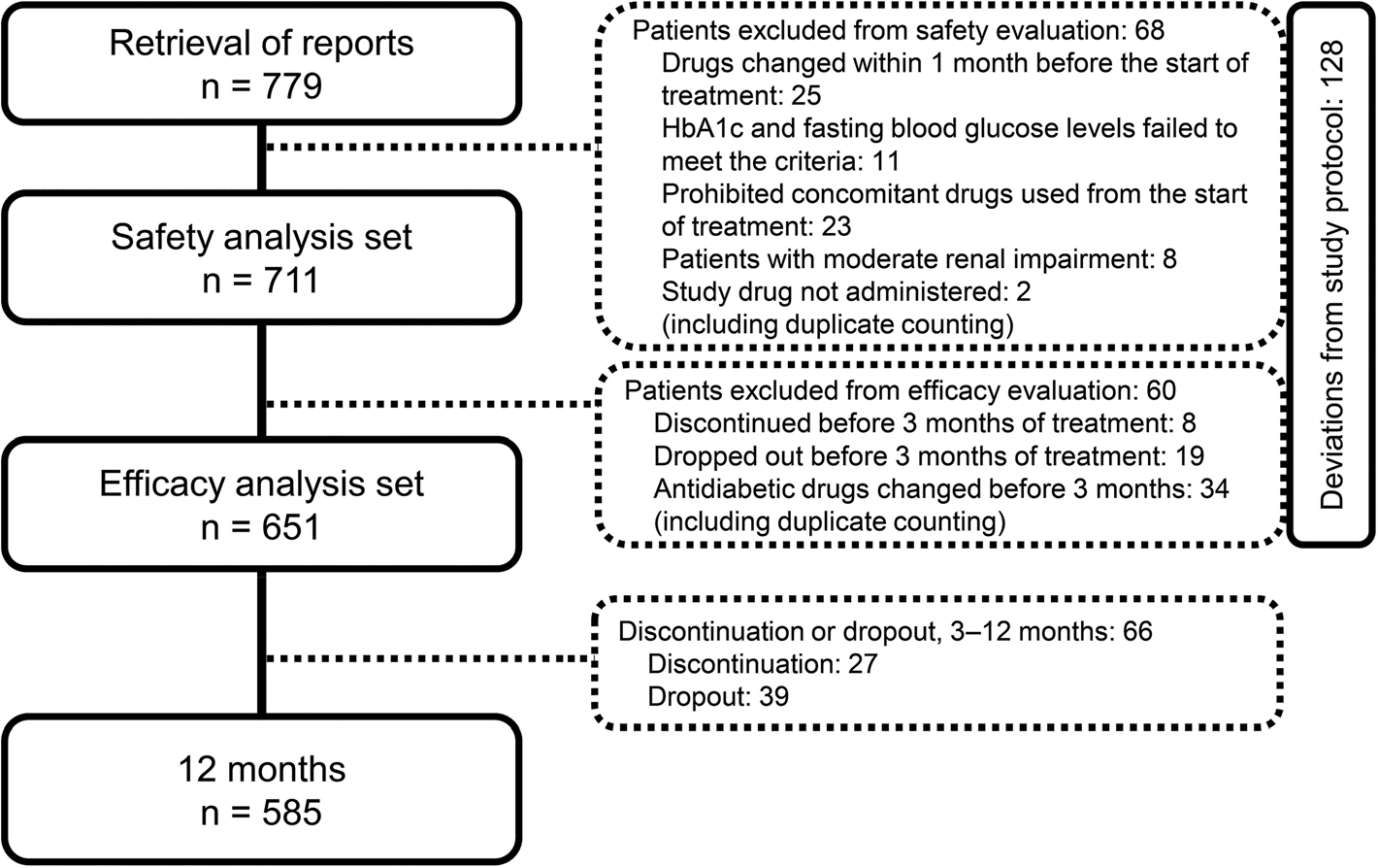
Patient enrollment flow diagram

Figure 2 shows the flow diagram of the patient enrollment in the study. Of the 779 patients with T2DM enrolled, 651 were included in the Efficacy analysis (Diet/exercise therapy, 189; Low-dose glimepiride, 72; Medium-dose glimepiride, 50; Biguanide, 99; Thiazolidine, 38; α-GI, 18; Combination therapy, 185). Table 1 shows the baseline demographics of the 651 patients who were classified according to the concomitant drug used and evaluated to determine treatment efficacy. Past or concurrent illnesses in the entire study population are also presented in Table 1. There were 22 (3.4%) participants at 3 months and 37 (5.7%) participants at 12 months reported as poor adherence of sitagliptin.

**Table 1:** Patient demographic characteristics

Group Parameter		(Sitagliptin only)	+Sitagliptin (combination therapy)					
	Overall	Diet/exercise therapy	Low-dose glimepiride (0.5–1.0 mg)	Medium-dose glimepiride (1.5–2.0 mg)	Biguanide	Thiazolidine	α-GI	Coadministration of two or more drugs
n	651	189	72	50	99	38	18	185
Age (y)	63.8 ± 11.8	64.6 ± 12.7	68.1 ± 11.1	65.3 ± 11.2	59.3 ± 11.4	64.3 ± 12.2	63.9 ± 11.7	63.2 ± 10.8
Sex (male %)	434 (66.7)	121 (64.0)	44 (61.1)	37 (74.0)	72 (72.7)	27 (71.1)	13 (72.2)	120 (64.9)
BMI (kg/m^2^)	25.2 ± 4.2	24.5 ± 4.1	24.3 ± 4.0	24.5 ± 3.5	25.8 ± 3.6	26.4 ± 7.1	25.9 ± 3.9	25.7 ± 4.1
Abdominal circumference (cm)	88.3 ± 11.1	87.3 ± 10.7	88.7 ± 10.7	85.1 ± 8.2	88.9 ± 9.0	92.9 ± 21.4	92.4 ± 7.2	87.9 ± 10.1
Disease duration (y)	8.8 ± 6.7	6.2 ± 5.7	8.2 ± 6.4	10.2 ± 8.1	9.4 ± 6.8	10.5 ± 7.1	8.7 ± 5.7	10.5 ± 6.5
Smoking habit (%)	143 (22.7)	32 (17.4)	14 (20.3)	16 (34.0)	26 (27.1)	5 (13.5)	4 (22.2)	46 (25.6)
Drinking habit (%)	301 (48.1)	79 (42.5)	31 (47.0)	33 (68.8)	51 (53.1)	19 (52.8)	10 (55.6)	78 (44.3)
HbA1c (%)	7.86 ± 1.07	7.67 ± 1.09	7.74 ± 0.86	8.23 ± 1.31	8.05 ± 1.10	7.64 ± 0.74	7.26 ± 0.76	8.00 ± 1.04
Converted mean HbA1c (mmol/mol)	62	60	61	66	64	60	56	64
Fasting blood glucose (mg/dL)	159.2 ± 41.5	152.7 ± 43.5	156.6 ± 35.4	166.5 ± 38.7	173.5 ± 48.1	154.1 ± 38.3	146.0 ± 43.3	158.9 ± 36.5
HOMA-IR	2.83 ± 1.80	2.36 ± 1.45	2.70 ± 1.69	2.97 ± 1.92	3.34 ± 1.72	2.68 ± 2.03	2.69 ± 1.75	3.01 ± 2.03
HOMA-β (%)	32.1 ± 27.1	32.6 ± 33.9	29.4 ± 20.1	33.4 ± 25.7	31.5 ± 22.7	29.1 ± 21.3	36.0 ± 35.2	33.0 ± 25.3
C-Peptide (ng/mL)	2.10 ± 0.89	2.10 ± 0.90	2.36 ± 1.64	2.08 ± 0.58	2.14 ± 0.74	1.97 ± 0.78	2.00 ± 0.74	2.05 ± 0.96
SBP (mmHg)	130.9 ± 14.9	128.9 ± 16.1	134.0 ± 13.4	131.8 ± 11.4	131.0 ± 16.5	131.6 ± 16.3	130.5 ± 13.4	131.4 ± 13.8
DBP (mmHg)	76.5 ± 10.5	75.7 ± 9.7	76.5 ± 9.5	77.3 ± 10.3	79.6 ± 11.9	76.8 ± 11.4	79.7 ± 12.0	75.1 ± 10.2
Hypertension	393 (60.4)	107 (56.6)	46 (63.9)	29 (58)	60 (60.6)	24 (63.2)	13 (72.2)	114 (61.6)
Dyslipidemia	417 (64.1)	102 (54)	41 (56.9)	34 (68)	72 (72.7)	25 (65.8)	12 (66.7)	131 (70.8)
Hyperuricemia	65 (10)	17 (9)	4 (5.6)	2 (4)	13 (13.1)	5 (13.2)	7 (38.9)	17 (9.2)
Retinopathy	48 (7.4)	7 (3.7)	3 (4.2)	2 (4)	18 (18.2)	1 (2.6)	3 (16.7)	14 (7.6)
Arteriosclerosis obliterans	55 (8.4)	8 (4.2)	2 (2.8)	2 (4)	25 (25.3)	2 (5.3)	2 (11.1)	14 (7.6)
Atrial fibrillation	16 (2.5)	5 (2.6)	2 (2.8)	2 (4)	2 (2)	1 (2.6)	0 (0)	4 (2.2)
Renal disease	49 (7.5)	5 (2.6)	2 (2.8)	2 (4)	16 (16.2)	3 (7.9)	4 (22.2)	17 (9.2)
Hepatic disease	56 (8.6)	15 (7.9)	3 (4.2)	4 (8)	14 (14.1)	4 (10.5)	3 (16.7)	13 (7)
Myocardial infarction	18 (2.8)	5 (2.6)	2 (2.8)	1 (2)	3 (3)	0 (0)	2 (11.1)	5 (2.7)
Cerebral stroke	45 (6.9)	8 (4.2)	4 (5.6)	3 (6)	6 (6.1)	3 (7.9)	4 (22.2)	17 (9.2)
Angina pectoris	27 (4.1)	9 (4.8)	2 (2.8)	3 (6)	6 (6.1)	1 (2.6)	1 (5.6)	5 (2.7)
Cardiac failure	11 (1.7)	3 (1.6)	3 (4.2)	1 (2)	1 (1)	0 (0)	0 (0)	3 (1.6)

Data presented as n (%) or mean ± SD

**Fig. 3:**
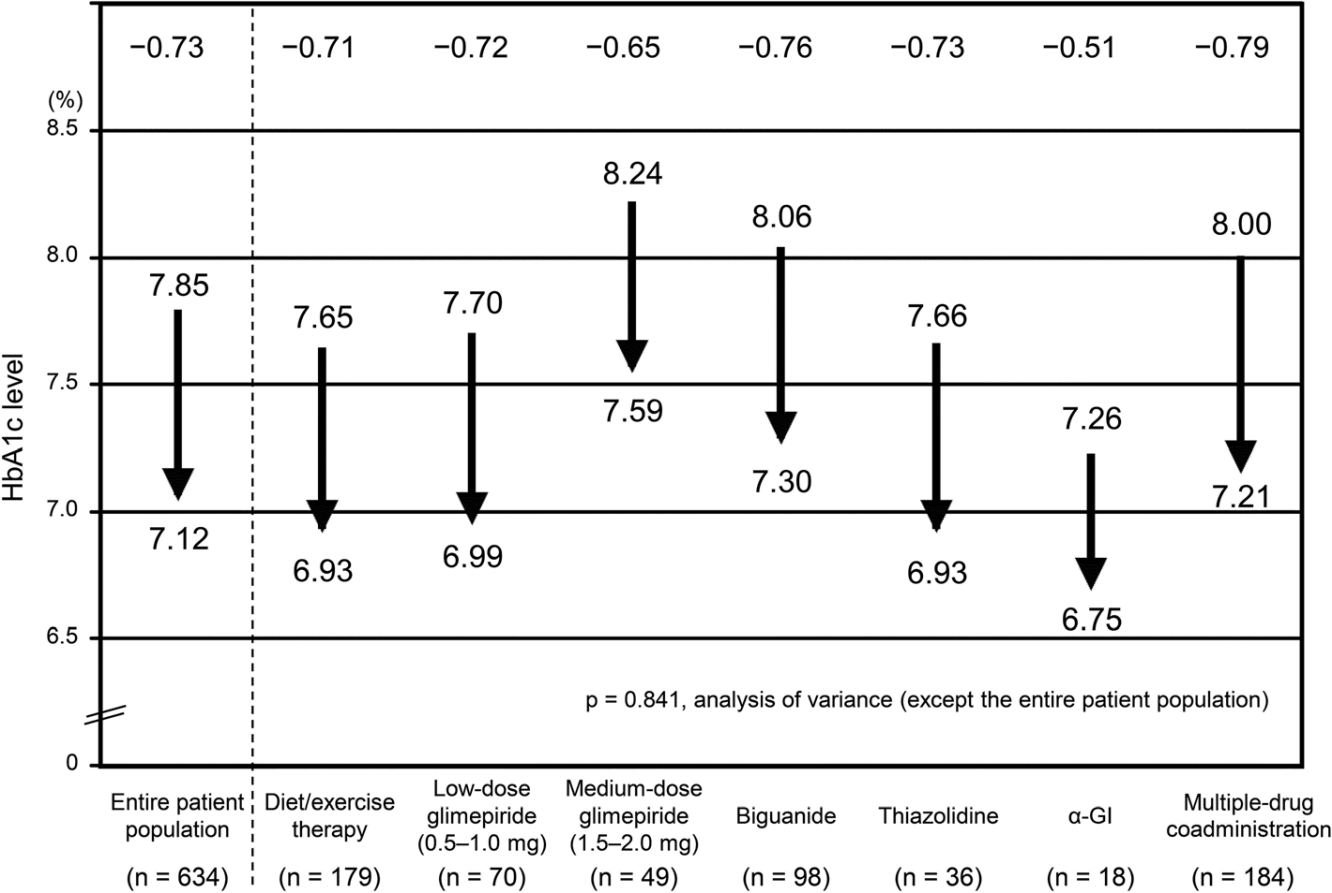
∆HbA1c level according to concomitant drug type (3 months)

The HbA1c level significantly decreased after one month of treatment compared to baseline (*p* < 0.05), and this reduction was maintained throughout 12 months of treatment (Additional flile 1: Figure S1). The change (95% CI) in HbA1c level from baseline in the entire patient population was −0.73% (−0.80 to −0.67) at 3 months of treatment. There was no significant difference in the change in HbA1c level between the patient groups treated with different concomitant drugs (Fig. 3 and Additional file 3).

The HbA1c level normalization rate, expressed as the percentage of patients who achieved an HbA1c level of <7.0% (53 mmol/mol), significantly increased at 1 month compared to baseline (*p* < 0.05), reaching an increase in 53.1% at 3 months (Additional flile 2: Figure S2). The fasting blood glucose normalization rate, calculated as the percentage of patients who achieved a fasting blood glucose level of <130 mg/dL, significantly increased at 1 month compared to baseline (*p* < 0.05), reaching an increase of 50.9% at 3 months (S2). We enrolled patients who meet the criteria of a glycated hemoglobin (HbA1c) level of ≥6.9% (52 mmol/mol) or a fasting blood glucose level of ≥ 130 mg/dL [6] during the observation period. Consequently, this study include patients who were achieved the HbA1c goal/or the fasting blood glucose goal at baseline.

Table 2 shows the changes (95% CI) in most laboratory parameters at 3 and 12 months after the start of treatment compared with baseline. Improvement was observed in many of the diabetes mellitus-related parameters.

**Table 2:** Laboratory test results

Parameter	n	0 month (mean ± SE)	n	3 months (mean ± SE) ∆3 months, mean (95% CI)	p	n	12 months (mean ± SE) ∆12 months, mean (95% CI)	p
HbA1c	649	7.86 ± 0.04	635 634	7.12 ± 0.04 −0.73 (−0.80, −0.67)	0.001>	577 576	7.11 ± 0.04 −0.69 (−0.76, −0.62)	0.001>
Converted mean HbA1c(mmol/mol)		62		54			54	
Fasting blood glucose	493	159.2 ± 1.9	446 413	135.1 ± 1.6 −26.3 (−29.7, −22.9)	0.001>	394 361	135.9 ± 1.7 −23.2 (−27.3, −19.2)	0.001>
HOMA-R	384	2.83 ± 0.09	367 318	2.71 ± 0.14 −0.36 (−0.51, −0.22)	0.001>	331 275	2.66 ± 0.11 −0.29 (−0.47, −0.10)	0.002
HOMA-β	382	32.1 ± 1.4	364 315	47 ± 3.2 11.5 (8.4, 14.6)	0.001>	324 270	43.9 ± 2 11.1 (7.6, 14.6)	0.001>
Fasting insulin	384	7.22 ± 0.21	367 318	8.09 ± 0.43 0.21 (−0.15, 0.57)	0.254	331 275	7.77 ± 0.29 0.29 (−0.10, 0.67)	0.147
1.5AG	254	6.4 ± 0.3	248 240	11.3 ± 0.4 4.8 (4.3, 5.2)	0.001>	213 207	10.2 ± 0.4 3.8 (3.2, 4.3)	0.001>
Glycoalbumin	254	21.1 ± 0.2	248 240	18.2 ± 0.2 −2.7 (−3.1, −2.4)	0.001>	213 207	18.5 ± 0.2 −2.5 (−2.9, −2.2)	0.001>
C-Peptide	200	2.1 ± 0.06	197 177	2.07 ± 0.06 0 (−0.11, 0.1)	0.925	152 137	2.17 ± 0.08 0.05 (−0.06, 0.15)	0.380
Proinsulin-to-insulin ratio	193	0.51 ± 0.02	187 169	0.45 ± 0.02 −0.05 (−0.08, −0.02)	0.033	150 135	0.49 ± 0.02 −0.01 (−0.05, 0.03)	0.857
CPI	200	1.38 ± 0.05	197 177	1.56 ± 0.05 0.23 (0.15, 0.32)	0.001>	152 137	1.66 ± 0.07 0.28 (0.18, 0.38)	0.001>
SBP	646	130.9 ± 0.6	635 634	129.3 ± 0.6 −1.7 (−2.9, −0.6)	0.004	576 575	130.2 ± 0.6 −0.3 (−1.6, 0.9)	0.587
DBP	646	76.5 ± 0.4	635 634	75.3 ± 0.4 −1.2 (−1.9, −0.4)	0.003	576 575	75.6 ± 0.4 −0.9 (−1.7, −0.1)	0.022
BMI	630	25.19 ± 0.17	601 599	25.24 ± 0.17 0 (−0.10, 0.05)	0.987	552 549	25.1 ± 0.18 −0.08 (−0.20, 0)	0.064
Triglycerides	589	149.3 ± 4.1	569 551	146.7 ± 5.1 −6.1 (−13.0, 0.7)	0.081	537 517	142.5 ± 4.2 −8.9 (−16.0, −1.3)	0.021
HDL-C	574	55 ± 0.6	555 537	53.8 ± 0.6 −1.1 (−1.8, −0.5)	0.001	527 506	53.6 ± 0.6 −1.6 (−2.3, −0.9)	0.001>
LDL-C	589	113.9 ± 1.2	563 545	110 ± 1.2 −3.2 (−4.9, −1.5)	0.001>	533 512	110.3 ± 1.2 −3.4 (−5.6, −1.2)	0.003

Upper row: Observed value (mean ± SE)

Lower row: Change (mean [95% CI])

*p*-value: versus 0 month, paired *t*-test

The factors that affect the blood glucose-lowering effect of sitagliptin were also investigated. A negative correlation (*R* = −0.528, *p* < 0.05) was observed between the baseline HbA1c level and the change in HbA1c level from baseline to 3 months (S3). Related factors other than baseline HbA1c level were also investigated. For this purpose, the approximate linear correlation between the baseline HbA1c level and that at 3 months of treatment was calculated. A single regression analysis was performed using the deviation from the correlation line as the dependent variable. The results indicate that age, body mass index, mean blood pressure and concomitant administration of medium-dose glimepiride significantly affected sitagliptin efficacy (Table 3). Using the factors that showed a significant effect, multiple regression analysis was performed on the deviation from the approximate linear correlation. Only medium-dose glimepiride significantly affected sitagliptin efficacy (*p* = 0.017; Table 3).

**Table 3:** Single and multiple regression analyses of baseline HbA1c and ∆HbA1c levels (at 3 months) using the deviation from the approximate linear correlation coefficient as the dependent variable

	Single regression analysis				Multiple regression analysis			
Independent variable	Regression coefficient	Lower	Upper	*P*-value	Partial regression coefficient	Lower	Upper	*P*-value
		Limit	Limit			Limit	Limit	
Age	0.006	0.002	0.011	0.008^‡^	0.004	−0.001	0.009	0.149
Sex (male)	−0.022	−0.140	0.096	0.715				
Smoking habit	0.015	−0.121	0.151	0.83				
Drinking habit	−0.037	−0.152	0.078	0.523				
Duration of diabetes mellitus	−0.004	−0.012	0.005	0.377				
Body mass index	−0.015	−0.028	−0.001	0.029^‡^	−0.009	−0.023	0.005	0.207
Mean blood pressure	−0.008	−0.013	−0.002	0.005^‡^	−0.004	−0.008	0.001	0.095
Diet/exercise group	0.097	−0.027	0.22	0.126				
Low-dose glimepiride group	0.054	−0.124	0.232	0.555				
Medium-dose glimepiride group	−0.264	−0.472	−0.056	0.013^‡^	−0.249	−0.452	−0.045	0.017^§^
Biguanide group	−0.073	−0.228	0.081	0.351				
Thiazolidine group	0.082	−0.159	0.323	0.504				
αGI group	0.028	−0.308	0.364	0.871				
Multi-drug coadministration group	−0.008	−0.131	0.115	0.9				

^‡^ *p* < 0.05, single regression analysis

^§^ *p* < 0.05, multiple regression analysis

Table 4 shows the changes in anti-diabetic medication during the study and 86 patients were increased the dose of sitagliptin after the 3 months of follow-up visits.

**Table 4:** Changes in antidiabetic medication during the study (After 3 months of follow-up) *n* = 585

Agent	Sulfonylurea	Biguanide	Thiazolidinedione	α-Glucosidase inhibitors	Sitagliptin
Patients who received additional antidiabetic agent(s)	26	18	9	2	0
Patients who increased dosage	8	26	0	0	86
Patients who decreased dosage	7	4	0	0	2
Patients who stopped receiving agent(s)	8	3	20	0	0

Adverse events observed in the 711 safety-evaluable patients included clinical symptoms in 37 patients and laboratory abnormalities in 18 patients (Table 5). 19 out of 55 patients discontinued administration of sitagliptin because of the adverse event. Four of these events, anemia (sitagliptin single-drug group), hypoglycemia (multiple-drug co-administration group, low-dose glimepiride co-administration group), urticaria (multiple-drug co-administration group), were judged by the investigator to be causally related to the study drug. The incidence of an adverse drug reaction was 0.56%. Hypoglycemia occurred in three patients (0.42%), but all the cases were mild. They were judged to be causally related to the study drug in one patient each in the multiple drug co-administration and low-dose glimepiride groups, and as possibly causally related to the study drug in one patient in the sitagliptin single-drug group.

**Table 5:** Summary of adverse events

Hypoglycemia	3
Hyperglycemia	1
Hematopoietic disorders	3
Cardiovascular disease	8
Respiratory disorders	2
Skin eruption	2
Gastrointestinal disorders	5
Hepatobiliary disorders	7
Renal and urinary disorders	2
Neuropsychiatric disorders	2
Others	20
Total	55

## Discussion

In the present study, the overall sitagliptin-induced decrease (95% CI) in HbA1c level from baseline was −0.73% (range, −0.80 to −0.67) at 3 months. In many studies, sitagliptin reportedly improved HbA1c and fasting blood glucose levels both as a single-drug therapy [3, 8–10] and as a combination therapy with other antidiabetic drugs [4, 5, 11]. In our study, we excluded patients undergoing insulin use, also some clinical trials [12, 13] reported about the combination therapy about sitagliptin and insulin. The patients in the present study had chronic diabetes mellitus, and many had already been inadequately controlled with antidiabetic drugs and additionally received sitagliptin. This suggests that the conditions under which this study was conducted were closer to routine clinical practice than those of another Japanese dose-finding study for sitagliptin [9]. Our study showed favorable, significant results for patients taking sitagliptin, which were similar to those obtained in other studies, despite the differences in study conditions.

In the present study, no significant difference in the change in HbA1c level was observed between the groups of patients who received different concomitant drugs. However, the extent of the improvement was greater in the patients with a higher baseline HbA1c level [S3]. This suggests the need to correct for the effect of the baseline HbA1c level when interpreting data obtained in studies with widely scattered baseline HbA1c values. Sitagliptin-induced improvement in the HbA1c level was more difficult to achieve in the medium-dose glimepiride group than in the other groups, based on the exploratory study on the factors that affect the blood glucose-lowering effect of sitagliptin, the multiple regression analysis of the correlation between the baseline HbA1c level and the change in HbA1c level from baseline based on the deviation from the approximate linear correlation (Table 3). The baseline HbA1c level in the medium-dose glimepiride group was high (8.24%), but the sitagliptin-induced improvement in HbA1c level failed to meet the expectation that the higher the baseline HbA1c level, the greater the improvement [14, 15]. Although there is no report stating that Glimepiride is likely to cause the secondary failure, in comparison with Sulfonylurea, Biguanide and Thiazolidinedione, it is reported that Sulfonylurea causes the secondary failure [2]. Although Glimepiride doesn’t beget the secondary failure easily in Sulfonylurea [16], compared to other drug groups in this study, it is also presumed that in Glimepiride middle-dose group, the pancreas may become exhausted. We can’t identify the cause of it because there were some patients who didn’t laboratory test. However the duration of Glimepiride middle-dose group was 10.2 years, it was longer than overall. Consequently, the effect of sitagliptin might not be obtained easily. By contrast, in a study where glimepiride was administered at a low or high dose and sitagliptin was added after dose reduction of glimepiride [17], sufficient efficacy was achieved in both groups. Patients in actual clinical settings have diverse treatment histories, which requires future studies on sitagliptin co-administration with medium- or high-dose glimepiride. Although, smoking and drinking rate are both high in the medium-dose glimepiride group. But we judged it is not significant elements in the multiple regression analysis.

To prevent complications, the target HbA1c level is <7.0% (53 mmol/mol) and the target fasting blood glucose level is 130 mg/dL for blood glucose control [6]. In the present study, the rate of achieving the target control level improved for both HbA1c and fasting blood glucose levels.

Laboratory test results showed that the homeostatic model of assessment (β index) significantly increased at 3 month after starting treatment compared with its baseline value, suggesting that sitagliptin enhances insulin secretion.

Patients with type 2 diabetes mellitus have reduced numbers of pancreatic β cells [18, 19]. In an animal experiment, sitagliptin reportedly had pancreatic β cell-protecting and growth-promoting effects [20], in our present study, the C-peptide reactivity index (CPI) significantly increased at 3 months after starting treatment. It was more likely because of improved beta-cell function rather than increased beta-cell mass because it was just 3 months after the start of sitagliptin, and considering this was an observation in human subjects. However, Nishimura et al. reported that CPI increased from baseline to 3, 6, 12, 18, and 24 months after the start of sitagliptin administration [21]. This indicated the pancreatic β cell-protective effect of sitagliptin in a clinical setting. Nishimura et al.also reported that greater CPI increase after sitagliptin administration were associated with the response to sitagliptin [21]. In our study, the number of patients who test CPI was limited. So we didn’t analysis CPI as the elements of logistic analysis. But CPI may be benchmark of the efficacy of Sitagliptin.

However, the insulin secretion-enhancing effect of glucagon-like peptide 1 is reportedly dependent on the blood glucose level [22]. A meta-analysis on the effect of DPP-4 inhibitors revealed that the hypoglycemic risk from DPP-4 inhibitors is similar to that from placebo or thiazolidines but lower than that from sulfonylurea drugs [23]. Sitagliptin did not induce excessive hypoglycemia [10], and in the present study, mild hypoglycemia occurred in only three patients.

DPP-4 inhibitors do not induce body weight increases [8, 24–26]. In the present study, sitagliptin did not cause any increase in the body mass index. Sitagliptin also decreases postprandial triglyceride level [27] and lowers blood pressure, blood lipid level [28] and alkaline phosphatase level [29]. Our present study also showed decreased systolic and diastolic blood pressures, decreased LDL-C level and decreased triglyceride levels, which is similar to results from previously published studies.

Incretin is secreted from the gastrointestinal tract by the stimulation of molecules such as glucose and fats that are produced after food is taken into the digestive organs. Thus, incretin enhances insulin secretion from the pancreatic β cells when the blood glucose level increases after a meal. By contrast, sitagliptin decreased both postprandial and fasting blood glucose levels [26]. In the present study, sitagliptin improved fasting blood glucose levels and the rate of achieving the target control level. Talk about adherence, Walker et al. reported 22% of DM patients are defined as poor adherence of medicine [30], but our study showed only 5.7% of poor adherence of sitagliptin.

We designed this study period because Iwamoto et al. reported that clinical treatment with sitagliptin for 12 weeks provided significant and clinically meaningful reductions in HbA1c. Although, if study drug doesn’t work enough, to fix medication for a long time is not good for patients. On the other hand, Nishimura et al. reported that a patient’s HbA1c change at 3 months may be a predictor of their HbA1c change at 24 months [11]. And our study also showed the HbA1c level of 12 month was similar to 3 month. According to the result of this study, we assume that 3 months of observation period is not too short to evaluate the clinical effects of sitagliptin.

### Study limitations

This was an open-label observational study (patients were not allocated to the groups), the number of patients were different in the each groups because we categorized by the type of pre-existing therapy for type 2 diabetes. In this study, we excluded participants undergoing insulin use and rapid-acting insulin secretagogues. Dose increases of sitagliptin, and additional administration, dose changes or discontinuation of other antidiabetic drugs were allowed starting after 3 months of treatment. This short duration is a limitations to assessing the long-term usefulness of sitagliptin administration. However, this study is significant in that it evaluated sitagliptin efficacy in patients treated with different concomitant drugs in actual clinical settings.

## Conclusion

Sitagliptin administration improved the HbA1c level and the rate of achieving the target control levels in patients with type 2 diabetes mellitus who were previously untreated with, or who were poorly responsive to, existing antidiabetic drugs. Thus, sitagliptin is expected to be effective for this patient group. Concomitant administration of sitagliptin to patients treated with medium-dose glimepiride only slightly improved blood glucose control after correction for the baseline HbA1c level.

## Additional files

Additional file 1: Figure S1. HbA1c level and changes over time. (TIF 160 kb)
Additional file 2: Figure S2. Rate of achieving the target fasting blood glucose level (HbA1c level). (TIF 268 kb)
Additional file 3: Correlation between baseline HbA1c level and change in HbA1c level after 3 months of treatment. (Strongly effective: Below the correlation line; Weakly effective: Above the correlation line). (TIF 113 kb)

## Abbreviations

95% CI: 95% Confidence interval
BMI: Body mass index
CPI: C-peptide reactivity index
DBP: Diastolic blood pressure
DPP-4: Dipeptidyl peptidase-4
HbA1c: Glycated hemoglobin
HDL-C: High-density lipoprotein cholesterol
LDL-C: Low-density lipoprotein cholesterol
MHLW: Ministry of Health, Labour and Welfare of Japan
NGSP: National Glycohemoglobin Standardization Program
SBP: Systolic blood pressure
SD: Standard deviation
SE: Standard error
TG: Triglycerides
α-GI: α-glucosidase inhibitors
